# Layered Sol–Gel Deposition of a Sn, Ti, Zn, and Pr Mixed Oxide Thin Film with Electrical Properties for Gas Sensing

**DOI:** 10.3390/gels9080638

**Published:** 2023-08-08

**Authors:** Izabella Dascalu, Cristian Hornoiu, Jose Maria Calderon Moreno, Petre Osiceanu, Simona Somacescu

**Affiliations:** “Ilie Murgulescu” Institute of Physical Chemistry—Romanian Academy, Splaiul Independentei 202, 060021 Bucharest, Romania; idascalu@icf.ro (I.D.); posic@icf.ro (P.O.); ssimona@icf.ro (S.S.)

**Keywords:** sol gel, SnO_2_, ZnO, TiO_2_, praseodynium, gas sensing

## Abstract

This article presents a layered mixed oxide thin film composed of Sn, Ti, Zn, and Pr obtained by sol–gel deposition for gas sensing applications. The film was characterized by X-ray diffraction (XRD), X-ray photoelectron spectroscopy (XPS), Raman spectroscopy, UV-Vis spectroscopy, Scanning electron microscopy with energy dispersive X-ray spectroscopy (SEM-EDX), and Electrochemical impedance spectroscopy (EIS). X-ray diffraction results showed the presence of a single crystalline phase with a cassiterite-like structure. Raman spectroscopy revealed characteristic bands of oxygen-deficient SnO_2_-based nanocrystallites. The band gap energy calculated from UV-Vis spectroscopy is Eg = 3.83 eV. The XPS proved the presence on the surface of all elements introduced by the inorganic precursors as well as their oxidation states. Thus, Sn^4+^, Ti^4+^, Zn^2+^, and Pr^3+^ were detected on the surface. Moreover, by XPS, we highlighted the presence of OH groups and water adsorbed on the surface. SEM showed the five-layer morphology of the film after five successive depositions. Electrochemical properties were determined by EIS-impedance spectroscopy. The selectivity for gas sensing was also investigated for methane, propane, and formaldehyde and the gas sensing mechanism was explained. The results indicated that the mixed oxide thin film exhibited high sensitivity and selectivity towards specific gases.

## 1. Introduction

Semiconductor oxides have great potential as basic materials in high-performance and modern technologies. Research in this field has led to the development of new materials with structures and characteristics of great interest that open up wide areas of applicability such as chemical engineering (nanopolymers, nanowires/nanotubes), optics (TCO materials, solar cells), optoelectronics (photodetectors, LEDs, lasers), electronics (varistors, transistors, transparent displays), medicine (diagnostic techniques, prostheses and implants), chemical sensors, piezoelectric devices, and other functional devices. These semiconductors have also attracted considerable attention because of their low cost, ease of use, and ability to detect a large number of gases.

Nanocrystalline semiconductor metal oxides with controlled composition are of great interest in the field of gas sensors. Recently, many papers have been reported regarding the combination of SnO_2_, SiO_2_, In_2_O_3_, CeO_2_, TiO_2_, and ZnO to obtain materials with structural, optical, electrical, photocatalytic, and sensing properties [1,2,3,4,5].

Rare earth oxides, such as Ce_2_O_3_, Pr_2_O_3_, and Tb_2_O_3_ do not have stable valence and therefore are rarely studied as gas sensors only, but for other applications in optics and photonics, electronics, and catalysis [6,7,8,9,10].

They can be used in small amounts as a doping material for various n-type semiconductors or as mixed oxides with n-type semiconductors. It could be that in combination with other oxides, the sensing properties of n-type oxide semiconductors mixed or doped with Pr_2_O_3_ are improved [11,12].

The powder of the mixed oxide of Sn, Ti, Zn, and Pr was shown to improve electrical properties by doping, and consequently Pr can increase the sensory performances of materials [1].

Materials based on metal oxides can be obtained by using the sol–gel method [13,14,15], one of the most widely used methods that offers the possibility of producing various materials with new, predefined properties in a simple process and at a relatively low cost.

The sol–gel method is versatile and allows control of material composition, morphology, and properties, making it very attractive for various applications, such as catalysts, sensors, optics, and ceramics. Recent studies show that this is a promising method for the deposition of gas sensors. Gas sensors obtained by the sol–gel method are used in a wide range of applications, including to monitor and detect hazardous gases such as CO, CO_2_, NO_2_, and CH_4_ [16,17,18] to ensure safety and air quality.

In this article, we attempted to obtain a sensor based on the previously studied composition (Sn:Ti:Zn:Pr molar ratios of 60:20:15:5) that exhibits a very good sensory response to CO. Considering the influence of the calcination temperature on the obtained electrical properties (the sensitivity to CO decreases with increasing calcination temperature), it was expected that a more sensitive material would be obtained at the treatment temperature of the film (450 °C). In this paper, we present the results of XRD, SEM-EDX, Raman, UV-Vis, XPS, and IS investigations used to study our film.

## 2. Results and Discussion

### 2.1. Structural and Morphological Properties

#### 2.1.1. XRD

**Figure 1:**
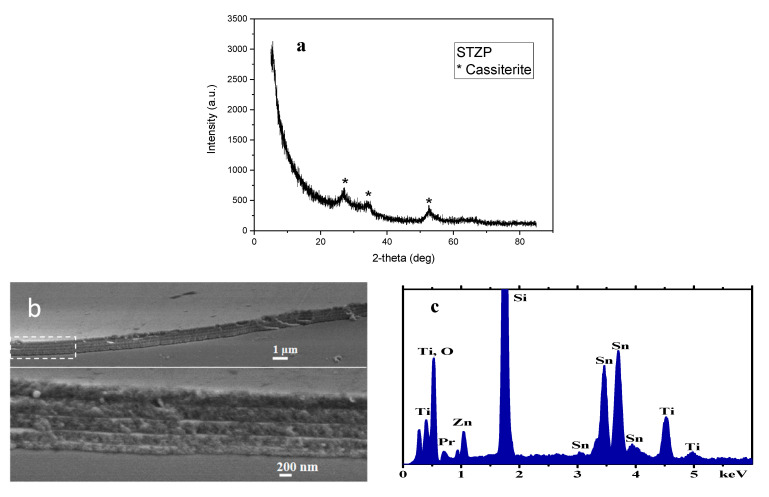
XRD pattern (**a**) SEM images at different magnification (**b**) and EDX spectrum (**c**) for STZP film.

The diffraction pattern for the Sn, Ti, Zn, and Pr mixed oxide thin film after heat treatment is shown in Figure 1a. The PDXL software from the Rigaku equipment, connected to the ICCD database, identified the cassiterite phase, characteristic of SnO_2_, with maximum diffraction peaks at 27.15°, 34.72°, and 52.42° matching with diffracting lines (110), (101), and (211) of SnO_2_ as tetragonal cassiterite (PDF 01-072-1147 [19]) as the single-component crystalline phase of the deposited STZP film. The crystal lattice planes appeared to shift towards higher 2-theta values, indicating a reduction in the lattice cell parameters (Table 1) due to the introduction of dopants in the cassiterite lattice, compared to standard lattice parameters of tetragonal SnO_2_ a = b = 4.737 Å, c = 3.185 Å.

The crystallite size was obtained using the Debye–Scherrer formula by direct measurement of the full width at half peak (2.85 deg) of the (211) peak at ~52° (Table 1).

#### 2.1.2. SEM-EDX

The cross-section SEM images of the STZP film using various magnifications reveal the film morphology. At low magnification, the film appears in the cross-section image with a flat surface with a uniform thickness of about one micron. The absence of any major defects or irregularities can be observed. Increasing the magnification level reveals details in the thin film’s cross-section. Distinct layers can be observed within the film, composed of five distinguishable layers with boundaries clearly visible at this magnification and thicknesses of the individual layers around 200 nm, increasing slightly from the first inner layer, close to the substrate, to the fifth, outer layer. EDX elemental analysis detects the film elements O, Pr, Sn, Ti, Zn, and Si element from the substrate. Measurements at different areas revealed a homogeneous composition of the film, with Sn:Ti:Zn:Pr cationic ratios of 60:20:15:5.

#### 2.1.3. Raman

**Figure 2:**
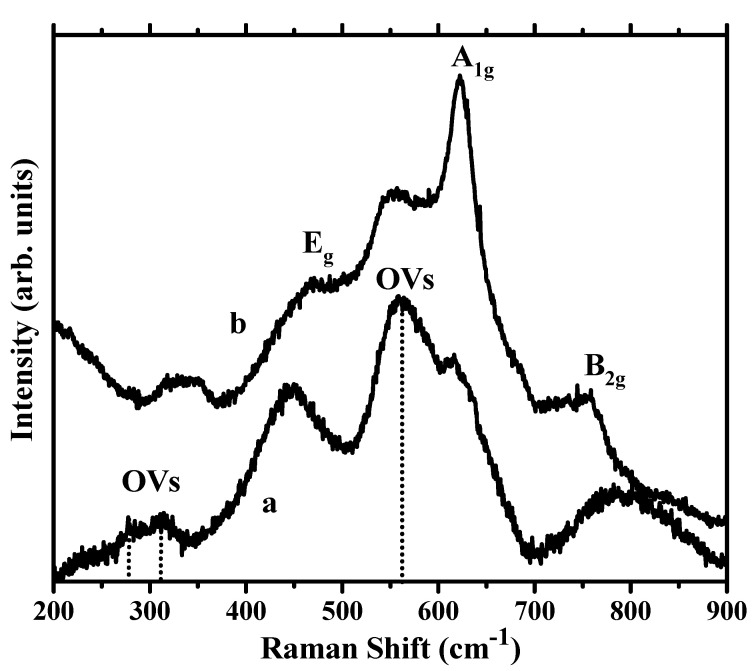
Raman spectra of the STZP film (**a**) and undoped SnO_2_ (**b**), showing intrinsic Raman modes of cassiterite and OVs induced modes (marked by dotted lines).

The Raman spectra of the STZP film based on the cassiterite lattice with significant substitution by lower valence cations (Zn^2+^, Pr^2+/3+^) can provide valuable information about the structural properties of the material. Cassiterite has several phonon modes that are typically observed in its Raman spectra. In the Raman spectrum of a cassiterite film with oxygen vacancies, these phonon modes will be present but may exhibit variations due to the structural changes caused by the vacancies. Group theory predicts four Raman-active modes for tin oxide with cassiterite structure. Several Raman bands are observed (Figure 2), out of which we can assign the one at 617 cm^−1^ to the A1g mode, the one at 450 cm^−1^ to the Eg mode, and the one at 780 cm^−1^ to the B2g mode [20,21,22,23].

However, additional Raman bands, marked with dotted lines in Figure 2, are not intrinsic Raman modes because they do not appear in bulk SnO_2_ according to symmetry analysis. The presence of oxygen vacancies (OVs) gives rise to these additional Raman modes that are characteristic of defect-related vibrations [24]. These additional modes appear as distinct peaks in the Raman spectrum and can provide specific information about the presence and nature of the vacancies. The most intense additional band observed at ~560 cm^−1^ in Figure 2 has been reported by several studies of nanosized SnO_2_ and assigned its origin to the activation of the Raman inactive A2g mode, related to the presence of OVs and local lattice disorder [24,25,26,27]. We attribute, therefore, the strongest observed mode at 560 cm^−1^ to surface in-plane OVs in cassiterite nanocrystallites, in agreement with previous studies. The presence of OVs can induce disorder and lattice strain into the cassiterite film, resulting in peak broadening in the Raman spectra. The very broad peaks observed in Figure 2 can be explained by the presence of oxygen vacancies induced by lower valence cations. We also observe a significant red shift in the characteristic A1g mode, from ~640 to 617 cm^−1^. Shifts in the peak positions of the Raman bands compared to a purely stoichiometric tin dioxide film also arise due to local lattice distortions or changes in the bonding environment caused by the vacancies [24,25,26].

Furthermore, an additional OVs related band is observed at ~315 cm^−1^. This band has been rarely observed in Raman studies of SnO_2_ nanocrystals. Density functional theory (DFT) calculations [26] reported that this band appears only in non-stoichiometric cassiterite with a certain density of OVs (SnO_2−x_) when the value of x is at least 0.06. We observe a broad split band extended towards lower frequencies, with a shoulder at 278 cm^−1^, a position of the OVs-induced peak at low frequencies that according to Li et al. appears at values of x over 0.20. Our results confirm Li’s calculations; considering the elemental composition of the film from EDX measurements (Sn_0.6_Ti_0.2_Zn_0.15_ Pr_0.05_O_2−δ_) and the cation valencies determined by XPS measurements, we estimated an average value of δ = 0.19 in our non-stoichiometric STZP film. Oxygen deficiency can cause a decrease in the ionic character of the Sn^4+^ state in the Sn-O bond and increases the density of the s-electron surrounding the Sn atom. This results in a decreased binding energy observed in the XPS spectra.

#### 2.1.4. UV-Vis Spectroscopy

Room temperature UV-Vis spectra of STZP film is shown in Figure 2.

In order to determine the band gap energy, the absorption coefficient (α) was calculated according to Lambert’s law which is indicated by the following relation:

α = (1/d) × ln(1/T), where T is the measured transmittance (%) and d—the thickness of the samples.

**Figure 3:**
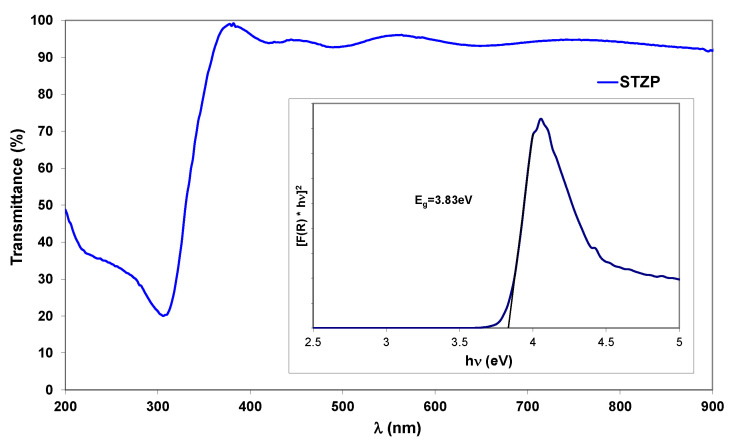
Room temperature UV-Vis spectra of STZP film annealed at 450 °C. The inset shows a corresponding optical band-gap plot.

The band gap energy was calculated using the Tauc method for direct band gap semiconductors. The linear region of the rising part of the curve [F(R) × hν]^2^ vs. hν was extrapolated to zero to obtain the band gap (Figure 3 inset), where hν is the energy of the photons. Tauc’s plot shows an indirect band semiconductor with E_g_ = 3.83 eV value similar to the literature value of the energy for SnO_2_ [28].

#### 2.1.5. XPS

X-ray Photoelectron Spectroscopy (XPS) analysis was performed to determine the elements present on the outermost surface layer (<10 nm) used as inorganic precursors, together with their corresponding chemical species. Thus, the high-resolution spectra for the most prominent transitions: Sn3d, Ti2p, Zn2p3/2, Pr3d5/2, and O1s were recorded.

**Figure 4:**
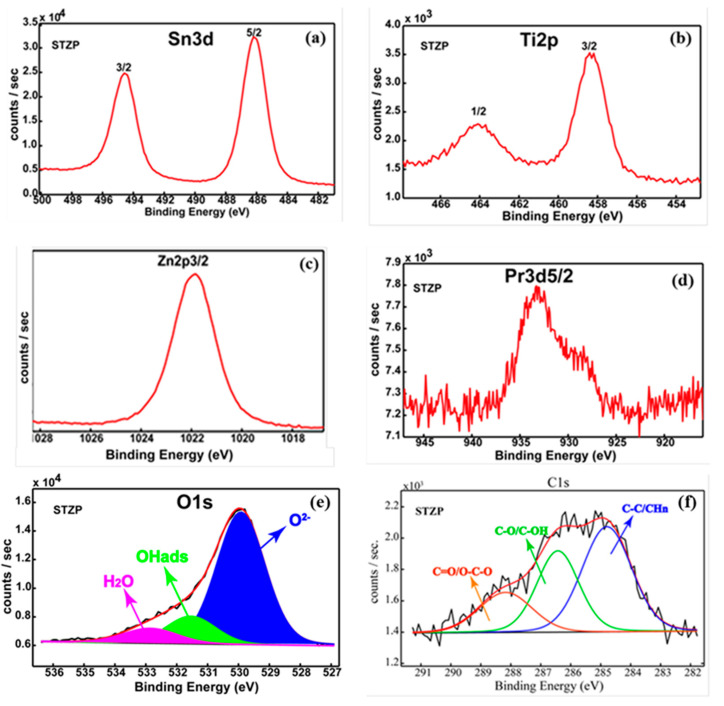
High-resolution spectra for the most prominent XPS transitions of the SZTP film Sn3d (**a**), Ti2p (**b**), Zn2p3/2 (**c**), Pr 3d5/2 (**d**), O1s (**e**), and C1s (**f**).

Sn was detected on the surface as Sn^4+^ at the binding energy (BE) ~486.1 eV (Figure 4a) It is worth mentioning that this value was shifted towards the lower BEs side as compared to the standard photoelecton line at ~487 eV. The chemical shift could be attributed to the lattice distortions and/or changes in the bonding environment caused by the presence of the lower valence cations which generate oxygen vacancies, as was highlighted by Raman Spectroscopy. Moreover, this finding is supported by our previous works [22]. Thus, we found that Sn3d_5/2_ is shifted towards lower BEs when cations such as In^3+^ and Cu^1+^ were incorporated in the SnO_2_ lattice. Ti was detected as Ti^4+^ (BE~458.3 eV), (Figure 4b).

In line with the aforementioned statement, the value of the Ti2p3/2 BE is lower in comparison with the standard one (~458.8 eV), beyond our experimental errors (±0.2 eV), suggesting that an interaction should take place inside the SnO_2_ lattice. We emphasize that the structural analysis does not reveal the formation of a secondary TiO_2_ phase; therefore, Ti is incorporated into the cassiterite structure as shown by XRD results. Zn and Pr were detected on the surface as Zn^2+^ (BE~1021.8 eV) (Figure 4c) and Pr^3+^ (BE~933.0 eV) (Figure 4d), according to NIST XPS Databases and Handbook of X-ray Photoelectron spectroscopy [29].

The oxygen chemistry (Figure 4e) displays the presence of the lattice oxygen (BE~530.0 eV—~71%), OH groups adsorbed on the top of the surface layer (BE~531.5 eV—~19%), and adsorbed H_2_O (BE~533.0 eV—~10%). The O1s spectrum clearly reveals a hydroxylated surface sample. One can notice that the hydroxylation degree of the surface is strongly affected by the synthesis protocols. Thus, our previous work highlighted that SnO_2_ and Pd/SnO_2_ sensors obtained by different chemical routes exhibit percentages of OH groups and H_2_O in the range of 20–40% [30,31]. Figure 4f shows the C1s deconvoluted spectrum, which exhibits three components attributed as follows: (1) C-C/CHn at 284.8 eV—used for callibration; (2) C–O/C–OH at 286.4 eV and (3) C=O, O=C–O at 288.3 eV [29]. We emphasize that cation relative concentrations after quantification from HR XPS spectra reveal the following atomic ratio: Sn:Ti:Zn:Pr—70.4:20.0:8.1:1.5 in agreement with EDX elemental analysis.

### 2.2. Electrical Properties Sensitivity Measurements of STZP Film

The bulk, the grain boundaries, and the electrode contributions to the overall resistance of the films can be resolved by exploiting the differences in their responses to an applied alternating current (AC) signal.

The software Z-view was used to fit the impedances of specific electrical circuits to the Nyquist experimental data (Nyquist representations) [1,13,14,32].

The electrical responses of the films were observed for different concentrations of CO (between 0 and 12,000 ppm). The gas sensing properties are characterized in terms of resistance and gas-sensing response. The resistance of the STZP film was decreased upon exposure to reducing gas (CO) and increased upon exposure to air, indicating that they are n-type semiconductors.

**Figure 5:**
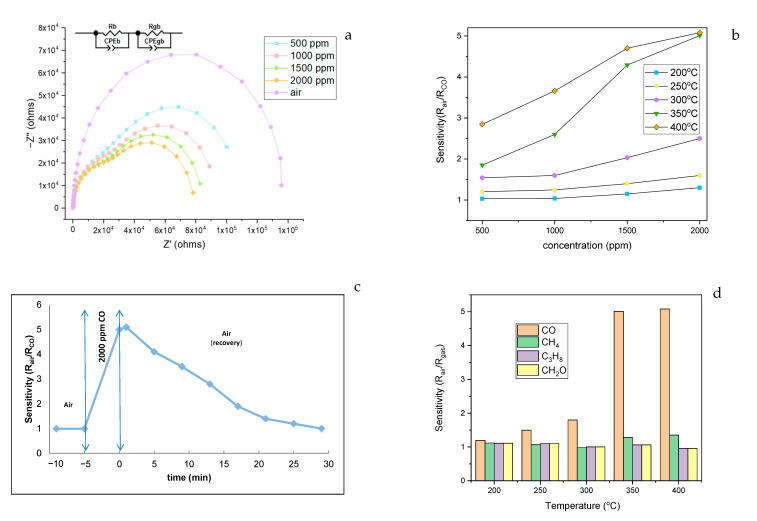
Impedance spectrum (Nyquist representation) frequency range 100 Hz to 1 MHz at 350 °C (**a**); sensitivity of STZP film at different CO concentrations (**b**); recovery time of STZP film at 350 °C for 2000 ppm CO in air (**c**); selectivity of STZP film to 2000 ppm (CO, CH_4_, C_3_H_8_) and 20 ppm (CH_2_O) (**d**).

The impedance spectroscopy measurements used to identify the electrical behavior of the STZP film were made in CO (in air) reducing environment, in the frequency range 100 Hz–1 MHz. Figure 5 shows the impedance spectra for samples at 350 °C in air and CO in air (500–2000 ppm). The observed two overlapping semicircles can be interpreted with the classical series circuit of two parallel resistance-constant phase elements (CPE).

In order to evaluate the sensitive properties as a sensor for the detection of CO, film tests were performed in the temperature range of 200–400 °C at different CO concentrations between 500 and 2000 ppm. The bulk resistance of the sample was determined from the interceptions of the first high-frequency arc with the real axis at low frequencies. However, with the increase in temperature, the grain boundary contribution decreased simultaneously.

For STZP film, the highest CO sensitivity (~5) was obtained for CO 2000 ppm concentrations at 350 and 400 °C, as shown in Figure 4b. The higher value of the sensitivity to CO of the film compared to the powder [1] is due to the lower treatment temperature of the mixed oxides.

Figure 4c shows the recovery time of the film at 2000 ppm CO and 350 °C. The response of the sensing film is the time needed to reach 90% of the resistance saturation value under exposure to the analyzed gas. The recovery time of the sensing film is the time needed to recover 10% of the original resistance value in air after exposure to the gas. The response for STZP film was found to be approximately 4 min and the recovery time was found to be approximately 20 min.

The correspondence of the semicircles obtained with the different regions of the sample, in this case the Nyquist representations, showed two semicircles for CO atmosphere [1]. The impedance parameters obtained by fitting experimental impedance data are listed in Table 2 (where Rgb is grain boundary resistance, Rb is bulk resistance, and CPE-T and CPE-P are constant phase parameters).

The sensor response strongly depends on the nature of the predominant defects involved in the conduction mechanism (mainly oxygen defects in oxide semiconductors).

#### 2.2.1. Selectivity Tests of STZP Film

To demonstrate selectivity (Figure 4d), the film was also tested with other pollutant gases, such as methane (CH_4_ 2000 ppm), propane (C_3_H_8_ 2000 ppm), and formaldehyde (CH_2_O 20 ppm).

The selectivity coefficients (SCO/Sgas the sensor response ratio between the target gas—CO and other gas) are presented in Table 3.

#### 2.2.2. Sensing Mechanism

**Figure 6:**
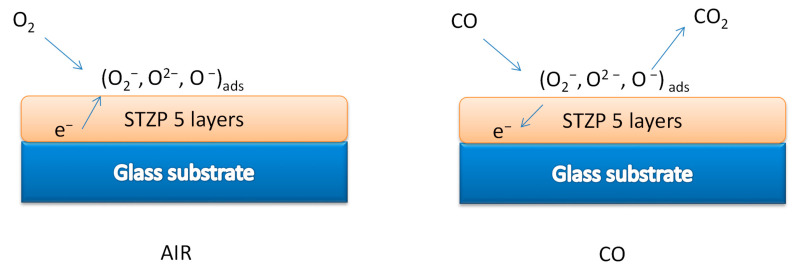
Intuitive schematic CO-sensing mechanism for STZP film.

Considering that the obtained film contains the highest proportion of SnO_2_, the mechanism is similar to this one, and is shown in Figure 6.

The adsorbed oxygen on the surface can be of several forms: O_2_, O_2_^−^, O^−^, and O^2−^. Of these species, O_2_ is quite inactive because its activation energy is high; its concentration is also very low. The sensing mechanism can be explained as follows. Stable oxygen ions species were O_2_^−^ below 200 °C, O^−^ between 200 and 300 °C, and O_2_^−^ above 300 °C [33].

The reactions of the oxygen species with CO molecules at different operating temperatures can be described using the following equations:

2CO + O_2_^−^ → 2CO_2_ + e^−^

CO + O^−^ → CO_2_ + e^−^

CO + O^2−^ → CO_2_ + 2e^−^

The carbon monoxide which is the reducing agent, reacts rapidly with O^−^ presented on the surface, but very slowly with O_2_^−^.

The n-type gas sensor changes its resistance (R) as the concentration of adsorbed oxygen changes. Adsorbed oxygen from clean air will be consumed in contact with carbon monoxide, with the resulting decrease in R being used to estimate the carbon monoxide concentration. The sensor recovers its original resistance level when the carbon monoxide is turned off.

The sensor response strongly depends on the nature of the predominant defects involved in the conduction mechanism of STZP film (mainly oxygen defects in oxide semiconductors).

## 3. Conclusions

In summary, by a sol–gel deposition method, we successfully synthesized a mixed oxide thin film based on SnO_2_ with Ti, Zn, and Pr incorporated in the lattice for enhanced CO sensing. The film was deposited by a spin-coating procedure on the glass substrate. Five layers were deposited and thermally treated at 280 °C after each step for consolidation. Finally, the thermal treatment was performed at 450 °C to obtain a crystalline film, as X-ray diffraction results confirmed.

The structural properties of the thin film obtained by XRD showed only the presence of SnO_2_-cassiterite, with a crystallite size of 6.4 nm.

SEM analysis showed five distinct layers with visible boundaries with individual layer thicknesses around 200 nm, and elemental EDX analysis detected the elements present in the film O, Pr, Sn, Ti, Zn, and the element Si in the substrate. The Raman spectra of the STZP film revealed the cassiterite lattice with significant substitution with lower valence cations (Ti^4+^, Zn^2+^, Pr^2+/3+^), agreeing with the structural properties of the material shown by XRD analysis. The band gap energy of the STZP film calculated from UV-Vis spectroscopy was Eg = 3.83 eV, which is similar to the value of SnO_2_.

The surface chemistry assessment, by XPS, highlighted the presence on the surface of all elements introduced from inorganic precursors. Sn^4+^, Ti^4+^, Zn^2+^, and Pr^3+^ were detected on the surface after processing the Sn3d, Zn2p3/2, and Pr3d5/2 HR spectra. Surface hydroxylation was also proved by XPS; OH groups adsorbed and water were revealed by deconvolution of the O1s singlet.

Electrical measurements showed that the best sensitivity of STZP film to carbon monoxide (CO) was ~5 at 350 °C, the response was ~4 min, and the recovery time was ~20 min. STZP film had a good selectivity for CO in the presence of other pollutant gases (CH_4_, C_3_H_8_, and CH_2_O).

The results of this study provide insight into the potential of the mixed oxide thin film as a gas sensor material for various applications.

## 4. Materials and Methods

In this work, we obtained a film based on Sn, Ti, Zn, Pr, through a modified sol–gel method in accordance with the synthesis presented in the article [1]. In this sol–gel synthesis route, the inorganic precursors Tin chloride (IV) anhydrous, Titanium propoxide (IV), Zinc acetate dehydrated, and Praseodymium acetylacetonate (III) hydrate were dispersed and stabilized by using Polyvinylpyrolidone (PVP360000). As template we used a nonionic surfactant Triton X100 and Tripropylamine (TPA) was added as a chelating agent.

The films based on Sn, Ti, Zn, and Pr mixed oxide were obtained by the spin-coating deposition method. Five layers were deposited on a glass substrate, with a consolidation heat treatment at 280 °C for 30 min for each deposited layer followed by a final treatment at 450 °C for 4 h. The films are called STZP.

X-ray diffraction (XRD) measurements were performed using a Rigaku Ultima IV-type diffractometer with Cu Kα radiation. The equipment operated at parameters of 40 kV and 30 mA, in the range of 15° < 2θ < 90°, being equipped with a device for measurements at low incidence angles ω = 0.5°.

The morphological analysis of the obtained films by scanning electron microscopy (SEM) was performed using a 3D FEI Quanta microscope, coupled with an energy-dispersive X-ray (EDX) spectrometer, at operating voltages of 5 kV, for secondary electron SEM images and 20 kV for elemental analysis EDX spectra.

Raman spectra were acquired in a Horiba Jobin–Yvon LabRam HR spectrometer with a He-Cd laser, using 325 nm as excitation wavelength. The power on the sample was kept below 0.5 mW to avoid sample damage due to irradiation. UV Raman spectra were collected using a 40× objective, in a UV-enhanced CCD camera and corrected by subtracting the background spectrum. The range of Raman shift recorded was between 200 and 700 cm^−1^ and the integration time of the measurements was 60 s. Ultraviolet-Visible (UV-VIS) Spectroscopy Diffuse reflectance spectroscopy was used to evaluate the optical properties and the band gap of STZP film. The spectra were recorded using a Perkin Elmer Lambda 35 spectrophotometer, in the range 200–1100 nm, with spectralon as a reference.

X-ray photoelectron spectroscopy (XPS) Surface analysis performed by X-ray photoelectron spectroscopy (XPS) was carried out on PHI Quantera equipment with a base pressure in the analysis chamber of 10-9 Torr. The X-ray source was monochromatized Al Kα radiation (1486.6 eV), while the unavoidable charging effect was minimized by using a dual beam (electrons and Ar ions) as a neutralizer. The Binding Energy (BE) scale was calibrated by C1s = 284.8 eV photoelectron line from the adsorbed environmental hydrocarbon on the surface (C–C or (CH)n bondings).

The electrical measurements were realized using a four-probe method AC impedance spectrometer with an electrochemical interface (Solartron 1260). The AC bias amplitude applied was 500 mV. Electrochemical Impedance spectra (EIS) were recorded in the frequency range of 1 MHz to 100 Hz from room temperature to 400 °C with a ProboStat cell (NorECs, Oslo, Norway).

The films were placed in a controlled atmosphere with a continuous gas flow of 177 mL/min (containing air and CO in He). Gas flows were provided by a calibrated system of mass flow controllers. Complex impedance measurements were used for the understanding of the mechanism of gas/solid interactions and the determination of the active regions in the films (surface, grain, and grain boundaries) that are involved in the detection of analyzed gas. The impedances Z = Z′ + j Z″ (Z′ and Z″ being, respectively, the real and imaginary components) were represented using Nyquist plots (Z″ vs. Z′).

## Figures and Tables

**Table 1:** Structural properties of the STZP film.

Phase	a (Å)	b (Å)	c (Å)	α = β = γ (°)	Crystallite Size (nm)
Cassiterite	4.70 (6)	4.70 (6)	3.11 (7)	90	6.4

**Table 2:** The fitted parameters related to impedance measurement at 350 °C.

CO (ppm)	R_gb_	CPE1-T	CPE1-P	R_b_	CPE2-T	CPE2-P
500	41,923	5.68 × 10^−10^	0.96204	57,931	1.38 × 10^−10^	1.272
1000	33,000	5.20 × 10^−10^	0.96767	44,720	1.29 × 10^−10^	1.298
1500	21,052	5.49 × 10^−10^	0.96591	42,905	1.26 × 10^−10^	1.319
2000	18,181	5.41 × 10^−10^	0.96777	38,829	1.30 × 10^−10^	1.307
air	80,123	4.71 × 10^−11^	1.253	44,574	7.79 × 10^−10^	0.948

**Table 3:** Selectivity coefficients.

Temperature (°C)	S_CO_/S_CH4_	S_CO_/S_C3H8_	S_CO_/S_CH2O_
200	1.07	1.15	1.08
250	1.40	1.40	1.36
300	1.83	2.23	1.79
350	3.12	4.40	3.76
400	3.55	5.48	4.99

## Data Availability

The datasets generated during and/or analyzed during the current study are available from the corresponding author on reasonable request.
